# Supplementary material for: Learning representations for image-based profiling of perturbations
Source: Nat Commun. 2024 Feb 21;15:1594. doi: 10.1038/s41467-024-45999-1 (PMC10881515; doi:10.1038/s41467-024-45999-1)
Supplement: Supplementary file 1 — Supplementary Information [file 41467_2024_45999_MOESM1_ESM.pdf]

Supplementary Information for “Learning representations for image-based profiling of perturbations”

Nikita Moshkov, Michael Bornholdt, Santiago Benoit, Matthew Smith, Claire McQuin, Allen Goodman, Rebecca A. Senft, Yu Han, Mehrtash Babadi, Peter Horvath, Beth A. Cimini, Anne E. Carpenter, Shantanu Singh, Juan C. Caicedo

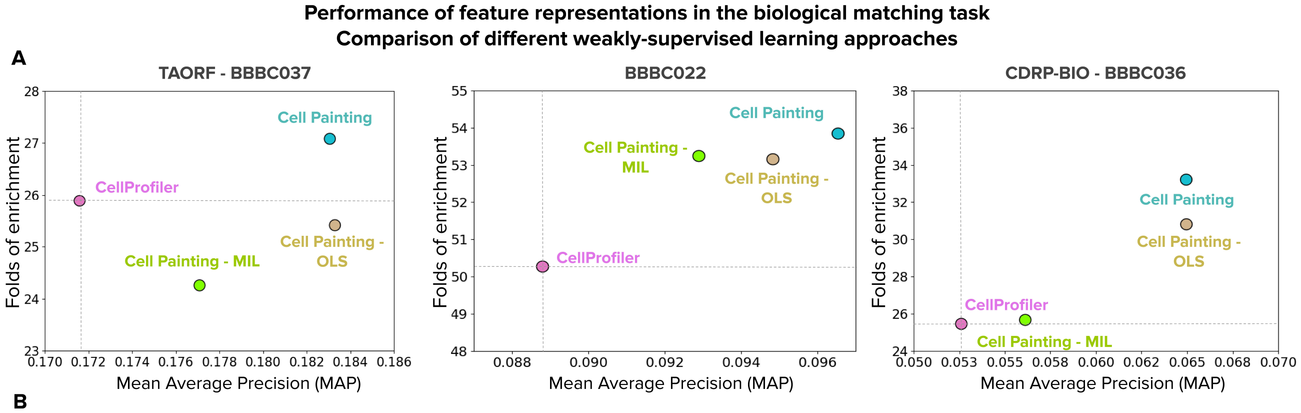

B

Experiment	Folds of Enrichment	Mean Average Precision
CellProfiler	25.8857	0.17159
Cell Painting - Online Label Smoothing (OLS)	25.4114	**0.18329**
Cell Painting - Multiple Instance Learning (MIL)	24.2556	0.17709
Cell Painting	**27.0820**	0.18306

Experiment	Folds of Enrichment	Mean Average Precision
CellProfiler	50.2667	0.0888
Cell Painting - Online Label Smoothing (OLS)	53.1579	0.09483
Cell Painting - Multiple Instance Learning (MIL)	53.2447	0.09289
Cell Painting	**53.8468**	**0.09654**

Experiment	Folds of Enrichment	Mean Average Precision
CellProfiler	25.4552	0.05261
Cell Painting - Online Label Smoothing (OLS)	30.7990	**0.06496**
Cell Painting - Multiple Instance Learning (MIL)	25.6677	0.05612
Cell Painting	**33.2222**	0.06495

Supplementary Figure 1. Evaluation of alternative weakly-supervised learning methods.
The three methods evaluated here differ in the loss function: 1-Cell Painting (cyan): regular classification loss with weak labels, 2-MIL [1] (green): multiple instance learning with attention, and 3-OLS [2] (brown): online label smoothing. The three methods train an EfficientNet model with the combined Cell Painting dataset. CellProfiler (pink) is used as a baseline result. The evaluation follows the same protocol described in the Methods section and reported in Figure 5. A) The performance of obtained feature representations is reported for the three benchmark datasets according to two metrics: Mean Average Precision (MAP) in the x axis and Folds of Enrichment in the y axis (see Methods). Each point indicates the mean of these metrics over all queries. B) The same information as in (A) in table format. Best performing result for each metric is highlighted with bold.

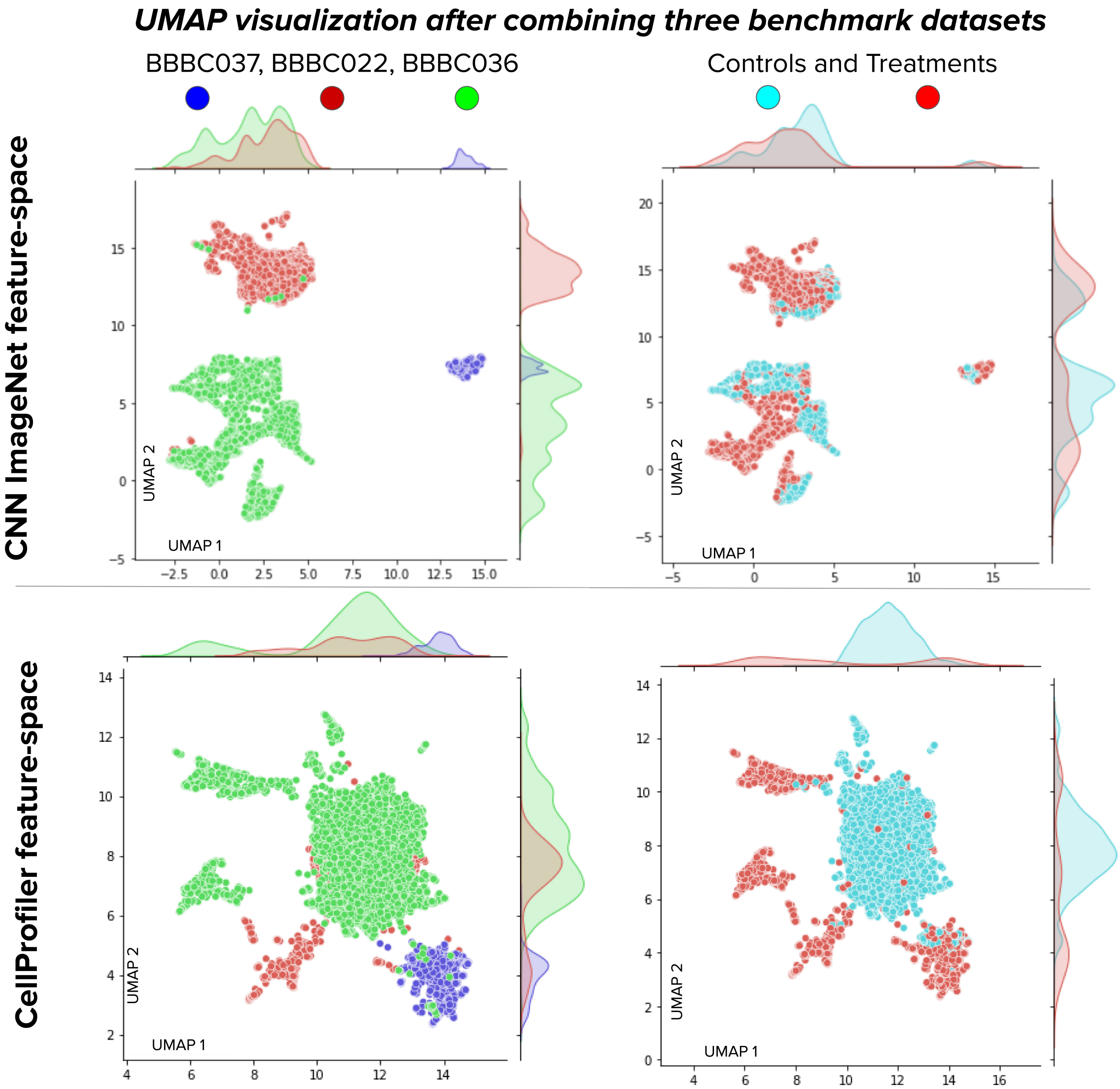

Supplementary Figure 2. UMAP visualization of well-level profiles after combining three benchmark datasets in CNN ImageNet and CellProfiler feature-spaces. *The top plots are colored by dataset (BBBC037 - blue, BBBC022 - red, BBBC036 - green). The bottom plots are colored by negative control (cyan) and treatments (red). This data was produced in preliminary experiments with dataset selection and shows the strongest treatments selected with use of corresponding features and Mahalanobis distance, instead of Euclidean in the main text. The CNN ImageNet features were selected with the penultimate layer. We see that different datasets are better integrated in the CellProfiler feature-space.*

Supplementary Note 1: Effect of masking single-cells with segmentation

Cell segmentation finds the spatial boundaries of each cell with respect to the rest of the cells in the same image. These boundaries can be used to isolate a single cell by zeroing out the pixels outside

the cell. This procedure, which we call “cell masking”, removes cell context and allows us to compute features that rely exclusively on the morphological content of the cell, rather than on any information provided by its neighbors or background. An advantage of masking cells is that it helps to minimize the impact of confounders such as cell density or noise, while paying attention to the structure of a single cell. However, segmentation algorithms are not perfect, and masks can cut parts of the cell out or completely miss entire cells altogether. In addition, masking may limit the information that can be extracted from cells, especially interactions with other cells, which is explicitly captured in classical feature extraction.

To assess the impact of single-cell masking in the biological matching task, a model was trained using the same setup as Cell Painting CNN, the only difference is that it was trained on masked single-cells and then feature extraction was also performed using masked single-cells. In all benchmarks, a CNN trained with masked cells generally underperforms the model trained with single-cell crops without masking with respect to the Folds of Enrichment metric. However, cell masking still performs better than the baseline models (CellProfiler and ImageNet) in terms of the mAP metric (Supplementary Figure 3). We recommend preserving *cells in context* as a more accurate strategy to profile the effect of treatments using Cell Painting.

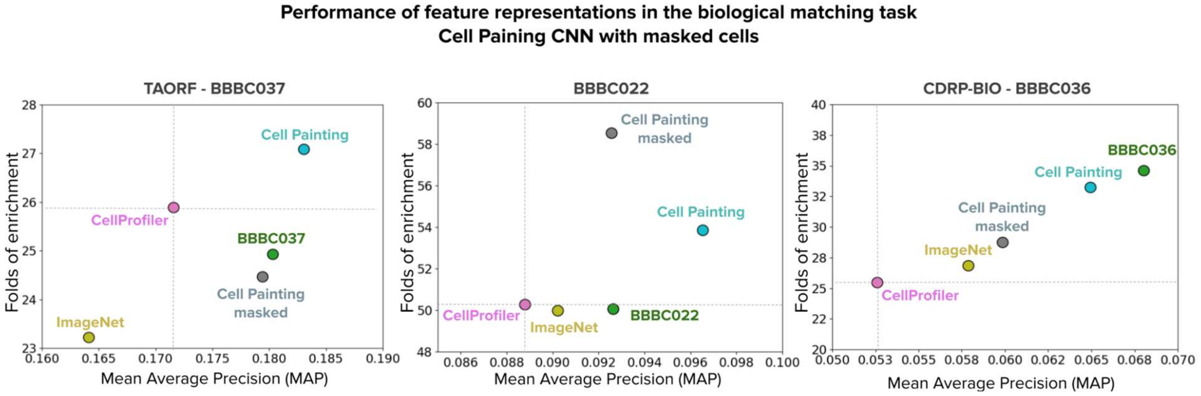

Supplementary Figure 3. Effect of training and feature extraction with masked single-cells. *The evaluation task is biological profile matching (see Figure 1G). Performance of feature representations for the three benchmark datasets according to two metrics: Mean Average Precision (MAP) in the x axis and Folds of Enrichment in the y axis (see Methods). Each point indicates the mean of these metrics over all queries using the following feature representations: CellProfiler (pink), a CNN pre-trained on ImageNet (yellow), a CNN trained on Cell Painting images from the same dataset (green), a CNN trained on the combined set of Cell Painting images (cyan) and a CNN trained on the combined set of masked Cell Painting images, benchmarked also on masked-out images (gray).*

UMAP visualization of mechanisms-of-action groups in chemical and phenotypic spaces

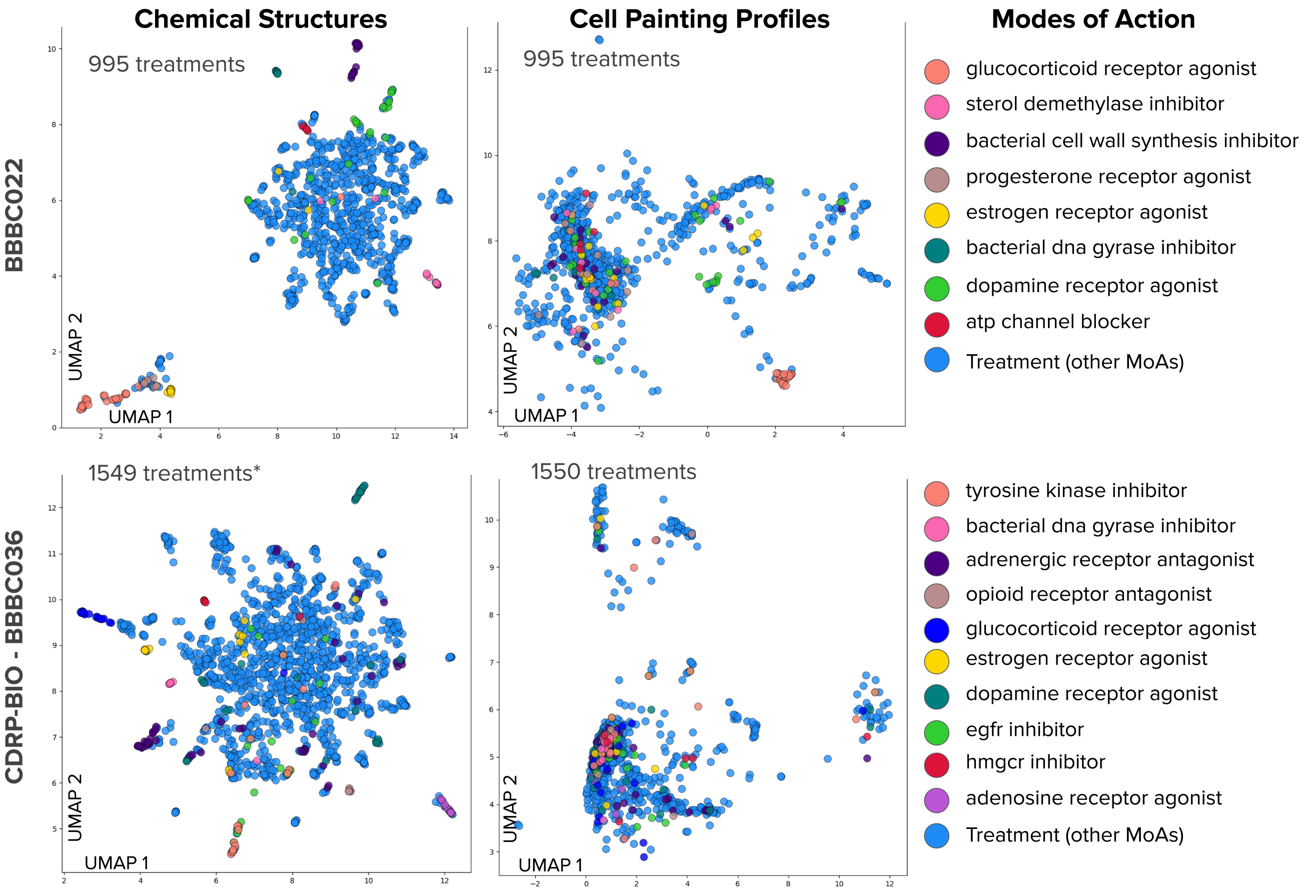

Supplementary Figure 4. UMAP visualization of mechanisms-of-action groups in chemical and phenotypic spaces. *Each point represents one compound treatment. Colors highlight groups of treatments with the same MoA. Left column: UMAPs of treatments in chemical feature space: Morgan fingerprints. Center column: UMAPs of treatments in phenotypic feature space (Cell Painting CNN 1). Right columns: legend of MoAs. Top row: BBBC022 dataset, bottom row: BBBC036 dataset.*

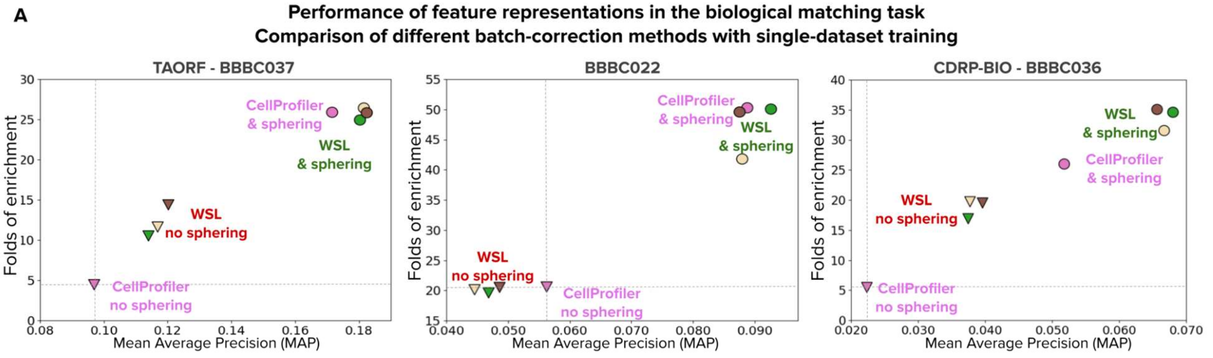

B

Experiment	Sph.	Folds of Enrichment	Mean Average Precision
CellProfiler	False	4.4477	0.09696
WSL	False	10.5120	0.11400
WSL & Harmony	False	13.7902	0.12164
WSL – GRL	False	11.62019	0.11688
CellProfiler	True	25.8857	0.17159
WSL	True	24.9248	0.18032
WSL & Harmony	True	25.8296	**0.18246**
WSL – GRL	True	**26.4155**	0.18154

Experiment	Sph.	Folds of Enrichment	Mean Average Precision
CellProfiler	False	20.5720	0.05626
WSL	False	19.5919	0.04685
WSL & Harmony	False	20.5011	0.04863
WSL – GRL	False	20.1100	0.04457
CellProfiler	True	**50.2667**	0.08880
WSL	True	50.0511	**0.09264**
WSL & Harmony	True	49.5692	0.08755
WSL – GRL	True	41.7768	0.08798

Experiment	Sph.	Folds of Enrichment	Mean Average Precision
CellProfiler	False	5.4510	0.02237
WSL	False	16.8798	0.03748
WSL & Harmony	False	19.5051	0.03962
WSL – GRL	False	19.7039	0.03775
CellProfiler	True	25.4552	0.05261
WSL	True	34.5929	**0.06803**
WSL & Harmony	True	**35.0619**	0.06566
WSL – GRL	True	31.5430	0.06672

Supplementary Figure 5. Evaluation of alternative batch-correction methods. *Two strategies are evaluated: 1-Harmony (brown) [3], and 2-Gradient reversal layer (yellow) (GRL) [4]. These two methods are used to learn corrected features with a Cell Painting CNN trained with WSL. Two additional baselines are added: CellProfiler (pink), and a standard Cell Painting CNN (green). Finally, all these strategies can be evaluated with sphering postprocessing (circles) or without sphereing correction (triangles). A) Performance of feature representations for the three benchmark datasets according to two metrics: Mean Average Precision (MAP) in the x axis and Folds of Enrichment in the y axis (see Methods). Each point indicates the mean of these metrics over all queries. B) The same information as in (A), but in table format. Best performing method for a metric is highlighted with bold. Sph. column indicates if sphering was used as a post-processing batch-correction step.*

Supplementary Note 2: Saliency Analysis

A saliency analysis of features was performed using the Grad-CAM algorithm. The goal of this experiment was to investigate if there are prominent features that characterize treatments or confounding factors. For this experiment we focused on an EfficientNet model trained with weakly supervised learning only on the BBBC022 dataset (leaving cells out), assuming that it is sensitive to technical variation specific to that dataset. We selected a subset of single cells from the validation set where the network prediction was correct, and then ran Grad-CAM to generate saliency maps that explain the classification. The layer used for analysis is block6a, which is where features are extracted from for downstream biological tasks. We started with cells from the DMSO control that come from different wells and plates and asked the question: does the model focus on the same structures to classify controls or are these specific to the plate or well? We also looked at treated cells from different plates for comparison (Supplementary Figure 6).

The saliency maps in all cases tend to concentrate attention in the central cell of the image, and sometimes attends a secondary cell or part of the context. There is no major or obvious difference between the images, and the saliency maps do not reveal a significant structure of interest. Unfortunately, Grad-CAM makes the assumption that features are localized in the 2D space, but the differences may be happening selectively in individual channels. Given how homogeneous the

saliency maps are across plates and treatments, we conclude that it is difficult to interpret features using this approach. We recommend using quantitative evaluations to objectively assess the performance of models, and to prevent drawing conclusions subjectively from a few examples.

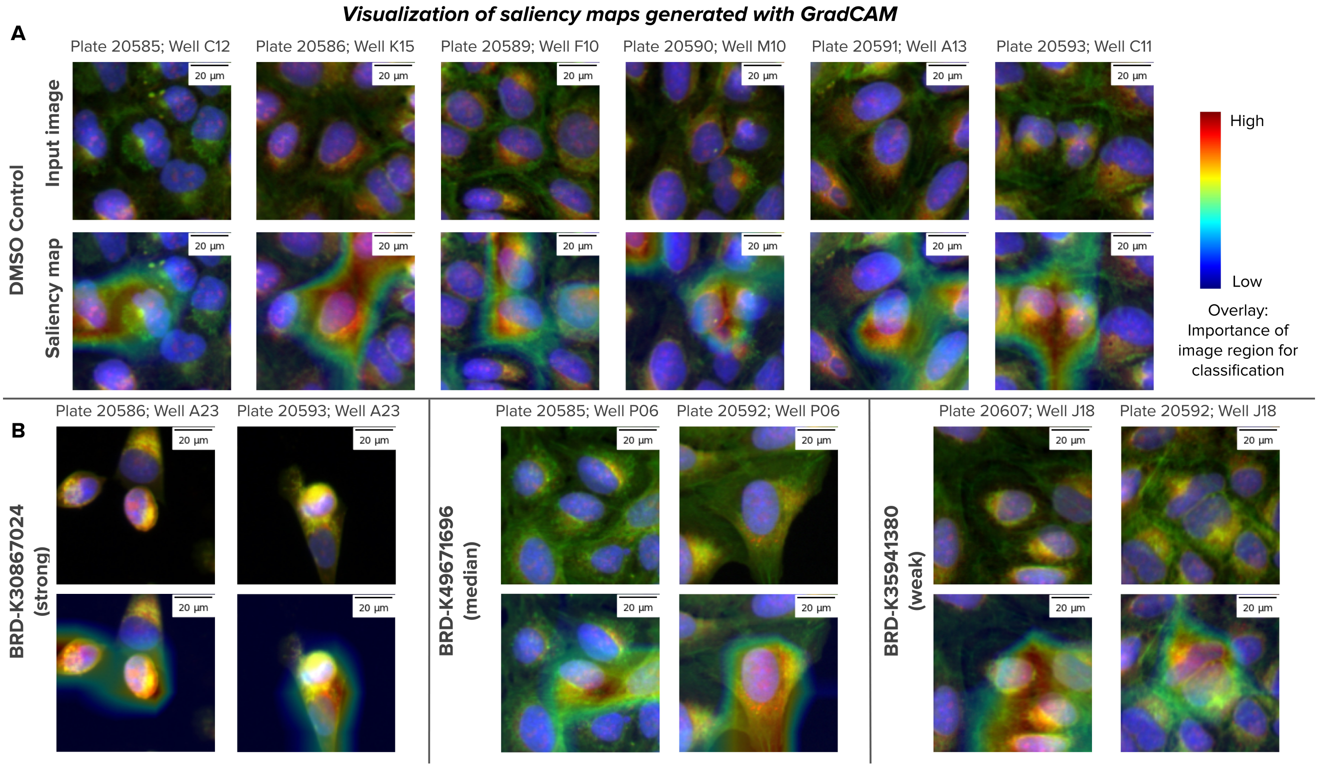

Supplementary Figure 6. Visualization of saliency maps generated with GradCAM. *Cell images are from the BBBC022 dataset, and the EfficientNet model was trained using weakly supervised learning on this dataset only. The saliency maps are calculated using block6a activations as the top layer, which is the layer used for feature extraction and downstream analysis. A) DMSO control cells representative of six plates (columns). The top row shows the input images, and the bottom row shows an overlay of the saliency map at 50% transparency over the input image. B) Cells treated with three treatments with strong, median and weak phenotypic response, all from the same MoA (serotonin receptor antagonist).*

Color overlay for cell images in both panels (see colorbar in panel A) depicts the importance of the image region for classifying the cell image to the true class.

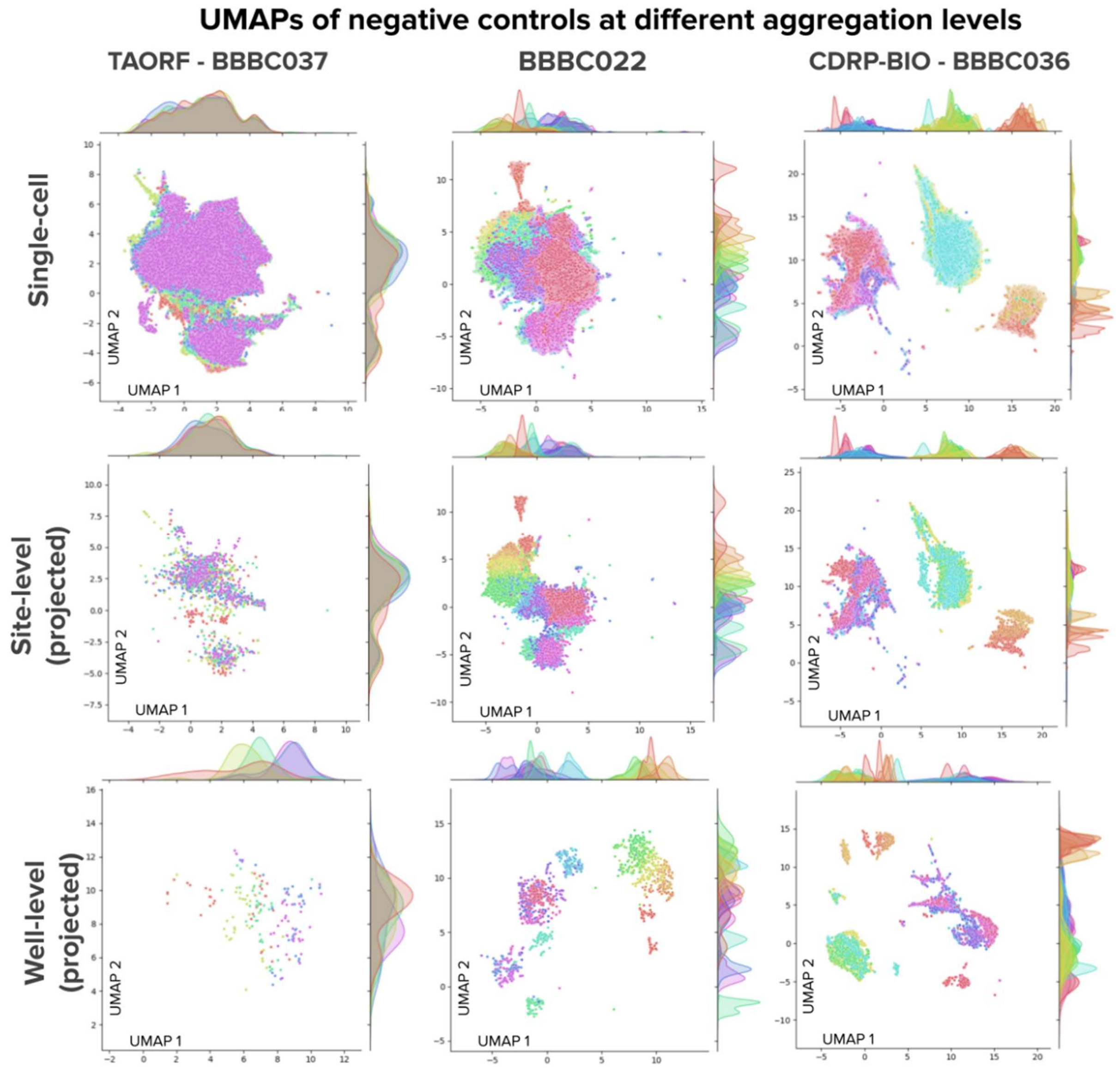

Supplementary Figure 7. UMAP visualization of different levels of data aggregation. *Features were extracted with single dataset experiments and leave-cells-out split (see Results and Methods) without any batch-correction. Only negative control (EMPTY or DMSO) single-cells\sites\well are depicted. Initial UMAP was computed for single-cell level, site-level and well-level profiles were then projected on single-cell UMAP space colored by plate IDs.*

Supplementary Note 3: Representations learned from Cell Painting images are computationally efficient

The dimensionality of the feature space of the Cell Painting CNN is also more compact than CellProfiler and the ImageNet CNN model (Supplementary Figure 8A), with the intermediate layer Conv6A of the EfficientNet B0 network being the best source of latent representations for downstream analysis in all of our experiments (Supplementary Figure 8B). This layer, after spatial average pooling, results in 672 features, compared to 1,700 of CellProfiler and 3,360 of ImageNet CNN (672 for each of the five imaging channels). The dimensionality of single-cell features has an impact on storage

space, especially for large scale experiments, making the Cell Painting CNN an efficient choice too (Supplementary Figure 8A).

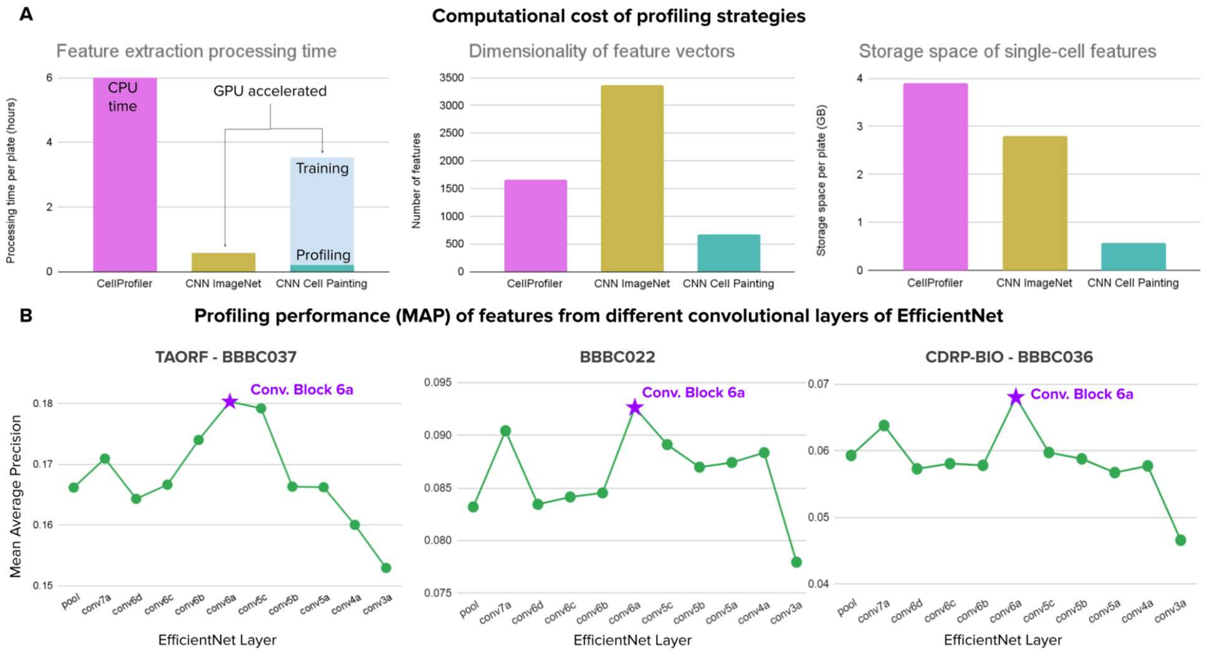

Supplementary Figure 8. Computational cost of profiling strategies. *Beyond improved accuracy and better performance in downstream tasks, our Cell Painting CNN is more computationally efficient than the baseline approaches. A) Computational cost in terms processing time per plate (in hours), dimensionality of representations (number of features), and storage space per plate (in GB), for the three representations evaluated in this work (x axes of plots). B) Downstream performance in the biological matching task from different convolutional layers of the EfficientNet model for all three datasets. We observe a consistent ability of Conv6A to yield better performance.*

Performance of feature representations in the biological matching task

A

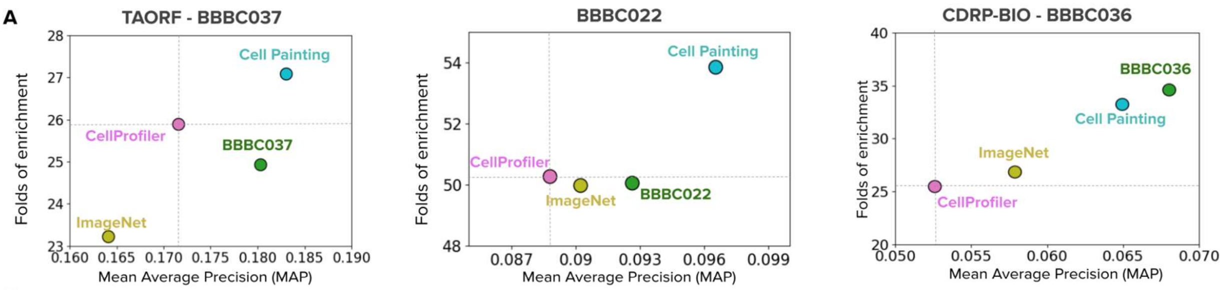

B

Experiment	Folds of Enrichment	Mean Average Precision
CellProfiler	25.8857	0.17159
ImageNet - Pretrained	23.2168	0.16413
WSL - Single dataset	24.9248	0.18032
WSL - Cell Painting CNN	**27.0820**	**0.18306**

Experiment	Folds of Enrichment	Mean Average Precision
CellProfiler	50.2667	0.08880
ImageNet - Pretrained	49.9737	0.09022
WSL - Single dataset	50.0511	0.09264
WSL - Cell Painting CNN	**53.8468**	**0.09654**

Experiment	Folds of Enrichment	Mean Average Precision
CellProfiler	25.4552	0.05261
ImageNet - Pretrained	26.8289	0.05788
WSL - Single dataset	**34.5929**	**0.06803**
WSL - Cell Painting CNN	33.2222	0.06495

Supplementary Figure 9. Quantitative evaluation of feature representations of treatment effects. *The evaluation task is biological profile matching (see Figure 1G). Panel A is the same as Figure 5A. A) Performance of feature representations for the three benchmark datasets according to two metrics: Mean Average Precision (MAP) in the x axis and Folds of Enrichment in the y axis (see Methods). Each point indicates the mean of these metrics over all queries using the following feature representations: CellProfiler (pink), a CNN pre-trained on ImageNet (yellow), a CNN trained on the combined set of Cell Painting images (cyan), and a CNN trained on Cell Painting images from the same dataset (green). In all cases, sphering batch-correction was applied on well-level profiles. B) The same information as in (A) in table format. Best performing result for each metric is highlighted with bold.*

Supplementary References

1. Ilse, M., Tomczak, J. & Welling, M. Attention-based Deep Multiple Instance Learning. in *Proceedings of the 35th International Conference on Machine Learning* (eds. Dy, J. & Krause, A.) vol. 80 2127–2136 (PMLR, 2018).
2. Zhang, C.-B. *et al.* Delving Deep Into Label Smoothing. *IEEE Trans. Image Process.* **30**, 5984–5996 (2021).
3. Korsunsky, I. *et al.* Fast, sensitive and accurate integration of single-cell data with Harmony. *Nat. Methods* **16**, 1289–1296 (2019).
4. Ganin, Y. & Lempitsky, V. Unsupervised Domain Adaptation by Backpropagation. in *Proceedings of the 32nd International Conference on Machine Learning* (eds. Bach, F. & Blei, D.) vol. 37 1180–1189 (PMLR, 07--09 Jul 2015).
