# Supplementary material for: Learning representations for image-based profiling of perturbations
Source: Nat Commun. 2024 Feb 21;15:1594. doi: 10.1038/s41467-024-45999-1 (PMC10881515; doi:10.1038/s41467-024-45999-1)
Supplement: Supplementary file 3 — Reporting Summary [file 41467_2024_45999_MOESM3_ESM.pdf]

Corresponding author(s): Juan C. Caicedo
juan.caicedo@wisc.edu

Last updated by author(s): Dec 20, 2023

Reporting Summary

Nature Portfolio wishes to improve the reproducibility of the work that we publish. This form provides structure for consistency and transparency in reporting. For further information on Nature Portfolio policies, see our Editorial Policies and the Editorial Policy Checklist.

Statistics

For all statistical analyses, confirm that the following items are present in the figure legend, table legend, main text, or Methods section.

n/a	Confirmed	
☐	☒	The exact sample size (*n*) for each experimental group/condition, given as a discrete number and unit of measurement
☐	☒	A statement on whether measurements were taken from distinct samples or whether the same sample was measured repeatedly
☐	☒	The statistical test(s) used AND whether they are one- or two-sided<br>*Only common tests should be described solely by name; describe more complex techniques in the Methods section.*
☒	☐	A description of all covariates tested
☒	☐	A description of any assumptions or corrections, such as tests of normality and adjustment for multiple comparisons
☐	☒	A full description of the statistical parameters including central tendency (e.g. means) or other basic estimates (e.g. regression coefficient) AND variation (e.g. standard deviation) or associated estimates of uncertainty (e.g. confidence intervals)
☒	☐	For null hypothesis testing, the test statistic (e.g. *F*, *t*, *r*) with confidence intervals, effect sizes, degrees of freedom and *P* value noted<br>*Give P values as exact values whenever suitable.*
☒	☐	For Bayesian analysis, information on the choice of priors and Markov chain Monte Carlo settings
☒	☐	For hierarchical and complex designs, identification of the appropriate level for tests and full reporting of outcomes
☒	☐	Estimates of effect sizes (e.g. Cohen's *d*, Pearson's *r*), indicating how they were calculated

Our web collection on statistics for biologists contains articles on many of the points above.

Software and code

Policy information about availability of computer code

Data collection	All the datasets used in this work are publicly available in the AWS Open Datasets - Cell Painting Gallery, which is accessible with the AWS Command Line Interface (CLI) v2. Data handling and processing was performed in the Linux Ubuntu OS. DeepProfiler was used to export single-cells from full images to create training datasets.

Data analysis	To run all the experiments in this study, we developed DeepProfiler, a tool for learning and extracting representations from high-throughput microscopy images using convolutional neural networks (CNNs). DeepProfiler uses a standardized workflow that includes image pre-processing, training of CNNs and feature extraction. DeepProfiler is implemented in Tensorflow 74 (version 2) and is publicly available on GitHub https://github.com/cytomining/DeepProfiler.<br><br>The DeepProfiler documentation (https://cytomining.github.io/DeepProfiler-handbook/) describes the steps for installing, configuring and running the software for profiling new images and for training models. In DeepProfiler we used the following EfficientNet implementation: https://github.com/qubvel/efficientnet. The processing and profiling pipelines for the three benchmarks evaluated in this work (Jupyter notebooks and Python scripts to analyze features) are available on GitHub: https://github.com/broadinstitute/DeepProfilerExperiments. This repository also includes the DeepProfiler configuration files used for training the models on each dataset, as well as the configuration for training the Cell Painting CNN model. In addition, the ground truth files and code for evaluation of the downstream tasks are also available in this repository. The Cell Painting CNN model (trained with leave-cells-out training-validation split) is available on Zenodo: https://doi.org/10.5281/zenodo.711455 7.<br><br>The ImageNet pre-trained EfficientNet model used in this study can be found here: https://github.com/Callidior/keras-applications/releases/download/efficientnet/efficientnet-b0_weights_tf_dim_ordering_tf_kernels_autoaugment.h5. Note, that this is an external resource that we used and it was not produced in this study.

For manuscripts utilizing custom algorithms or software that are central to the research but not yet described in published literature, software must be made available to editors and reviewers. We strongly encourage code deposition in a community repository (e.g. GitHub). See the Nature Portfolio guidelines for submitting code & software for further information.

Data

Policy information about availability of data

All manuscripts must include a data availability statement. This statement should provide the following information, where applicable:

- Accession codes, unique identifiers, or web links for publicly available datasets
- A description of any restrictions on data availability
- For clinical datasets or third party data, please ensure that the statement adheres to our policy

The Cell Painting CNN-1 model (trained with leave-cells-out training-validation split) is available on Zenodo: https://doi.org/10.5281/zenodo.7114557.
The Cell Painting datasets (raw images and CellProfiler profiles) are available at public S3 buckets:
BBBC037 gene overexpression dataset in U2OS cells s3://cytodata/datasets/TA-ORF-BBBC037-Rohban/profiles_cp/TA-ORF-BBBC037-Rohban/

BBBC022 compound screening in U2OS cells
s3://cytodata/datasets/Bioactives-BBBC022-Gustafsdottir/profiles/Bioactives-BBBC022-Gustafsdottir/

BBBC036 compound screening in U2OS cells s3://cytodata/datasets/CDRPBIO-BBBC036-Bray/profiles_cp/CDRPBIO-BBBC036-Bray/

BBBC043 gene overexpression dataset in A549 cells
s3://cytodata/datasets/LUAD-BBBC043-Caicedo/profiles_cp/LUAD-BBBC043-Caicedo/

LINCS compound screening in A549 cells
s3://cellpainting-gallery/cpg0004-lincs/broad/images/2016_04_01_a549_48hr_batch1/

The combined Cell Painting dataset which was collected using the above datasets is available at Cell Painting gallery https://github.com/broadinstitute/cellpainting-gallery/tree/main under accession cpg0019-moshkov-deepprofiler (S3 bucket). The single-cell embeddings extracted with Cell Painting CNN-1 model, aggregated well-level and treatment-level profiles and the metadata used for profiling of the benchmark datasets will be available in the same S3 bucket.

Human research participants

Policy information about studies involving human research participants and Sex and Gender in Research.

Reporting on sex and gender	No human participants were involved in the study.
Population characteristics	No human participants were involved in the study.
Recruitment	No human participants were involved in the study.
Ethics oversight	No human participants were involved in the study.

Note that full information on the approval of the study protocol must also be provided in the manuscript.

Field-specific reporting

Life sciences study design

All studies must disclose on these points even when the disclosure is negative.

Sample size	The results are reported in three benchmark datasets, each corresponds to one high-content screening experiment, after data filtering the sample sizes (used for training and evaluation of the models) are the following:<br>BBBC037: 205 treatments placed in 1029 wells. Additionally, there are 175 negative control wells. In total: 1024 wells.<br>BBBC022: 995 treatments placed in 3971 wells. Additionally, there are 1280 negative control wells. In total: 5251 wells.<br>BBBC036: 1550 treatments placed in 12180 wells. Additionally, there are 3528 negative control wells. In total: 15 708 wells.<br><br>We selected these three datasets for the study because each of them is a large collection of perturbations of two types: chemical and genetic perturbations. This covers a sufficient space of phenotypic responses for studying representation learning for cell morphology.
Data exclusions	We conducted quality control of images in all the three datasets by analyzing image-based features with principal component analysis. The outliers observed in the first two principal components were flagged as candidates for exclusion, and were visually inspected to confirm rejection. We found most of these images to be noisy or empty and not suitable for training and evaluation. With this quality control, two wells were removed from BBBC037, 43 wells from BBBC022, and no wells were removed from BBBC036. If treatments had multiple concentrations in BBBC022 and BBBC036, we kept only the maximum concentration for further analysis and evaluation.<br>The metadata used for the extraction of features will be made publicly available.
Replication	Treatments (also refereed to as perturbations) initially had five replicates (treatment in a single well in five different plates) in BBBC037 dataset, four replicates in BBBC022 dataset and up to eight replicates in BBBC036 dataset.<br><br>Multiple replicates are standard in high-throughput imaging studies to measure signal strength and replicability. In average, the three datasets exhibited more than 80% consistency in replicability with approximately 50% of the treatments having a phenotype significantly different from control samples. This level of consistency and replicability is sufficient for investigating the effects of treatments and for investigating methods that can amplify that signal.
Randomization	Treatments were not randomly allocated in experimental plates, but followed the specific predefined plate layout.
Blinding	Blinding is not applicable for this research, mainly because it is a retrospective study. The compound and gene overexpression screens were designed in previous studies, and our goal was to investigate if it was possible to extract more signal from these datasets given what it is currently known about these treatments (mechanism of action annotations).

Reporting for specific materials, systems and methods

We require information from authors about some types of materials, experimental systems and methods used in many studies. Here, indicate whether each material, system or method listed is relevant to your study. If you are not sure if a list item applies to your research, read the appropriate section before selecting a response.

Materials & experimental systems

n/a	Involved in the study	
☒	☐	Antibodies
☐	☒	Eukaryotic cell lines
☒	☐	Palaeontology and archaeology
☒	☐	Animals and other organisms
☒	☐	Clinical data
☒	☐	Dual use research of concern

Methods

n/a	Involved in the study	
☒	☐	ChIP-seq
☒	☐	Flow cytometry
☒	☐	MRI-based neuroimaging

Eukaryotic cell lines

Policy information about cell lines and Sex and Gender in Research

Cell line source(s)	U2OS - female, A549 - male.
Authentication	None of the cell lines used were authenticated.
Mycoplasma contamination	Cell were not tested for mycoplasma contamination.
Commonly misidentified lines<br>(See ICLAC register)	None.
